# A confirmed severe case of human infection with avian-origin influenza H7N9: A case report

**DOI:** 10.3892/etm.2014.2159

**Published:** 2014-12-30

**Authors:** HUI-FANG CAO, ZHONG-HUI LIANG, YING FENG, ZI-NAN ZHANG, JING XU, HE HE

**Affiliations:** 1 Department of Respiratory Medicine, Jingan District Centre Hospital of Shanghai, Jingan Branch of Huashan Hospital Affiliated to Fudan University, Shanghai 200040, P.R. China; 2 Department of Radiology, Jingan District Centre Hospital of Shanghai, Jingan Branch of Huashan Hospital Affiliated to Fudan University, Shanghai 200040, P.R. China

**Keywords:** fever, infection, hypoxia, human infection with the H7N9 avian influenza

## Abstract

A male patient, aged 77 years, was admitted to hospital with the chief complaint of persistent hyperpyrexia that had presented for four days. The patient also suffered from hypoxemia, and a large white shadow in the left lung was observed on a chest radiograph, indicating inflammation. No therapeutic effect was observed with anti-infection treatment. The patient admitted a history of direct contact with live chickens two weeks prior to hospital admission. The day after admission to the Jingnan District Centre Hospital of Shanghai (Shanghai, China), the patient was diagnosed with severe H7N9 avian influenza infection by nasopharyngeal swab and blood sampling detection. Although the patient received anti-infective drugs, intubated assisted ventilation and circulation support, the condition of the patient continued to rapidly deteriorate. Oxygen saturation decreased and gastrointestinal bleeding occurred, with the body temperature fluctuating between 39 and 40°C. By day 6 after admission, the patient presented with circulatory failure, with liver and renal failure. On day 7, the blood pressure of the patient was unable to be measured, and the patient was diagnosed with multiple organ dysfunction. Subsequently, clinical death was declared with the patient exhibiting asystole and no spontaneous breathing.

## Introduction

Human infections with H7N9 usually occur following recent exposure to poultry, which causes upper respiratory tract disease to progress into pneumonia and subsequently multiple organ failure (1). A diagnosis primarily depends on virus detection using nasopharyngeal swabs and blood sampling (2). Early diagnosis may be achieved through questioning into the epidemic history; an epidemic of influenza can be easily confounded with seasonal flu, and requires differentiation with pneumonia through careful observation of lung disease progression and the general condition of the patient. Laboratory investigations comprise various methods, including reverse transcription quantitative polymerase chain reaction (RT-qPCR), viral isolation and full-genome sequencing, all of which are able to confirm whether the patient is infected with the novel H7N9 virus (3).

In the present study, the patient exhibited a rapid deterioration; however, a diagnosis of H7N9 avian influenza was only confirmed after five days of continuous fever. Thus, the treatment process has provided experience for dealing with cases of H7N9, particularly for community health centers.

## Case report

### Clinical presentations

**Figure 1:**
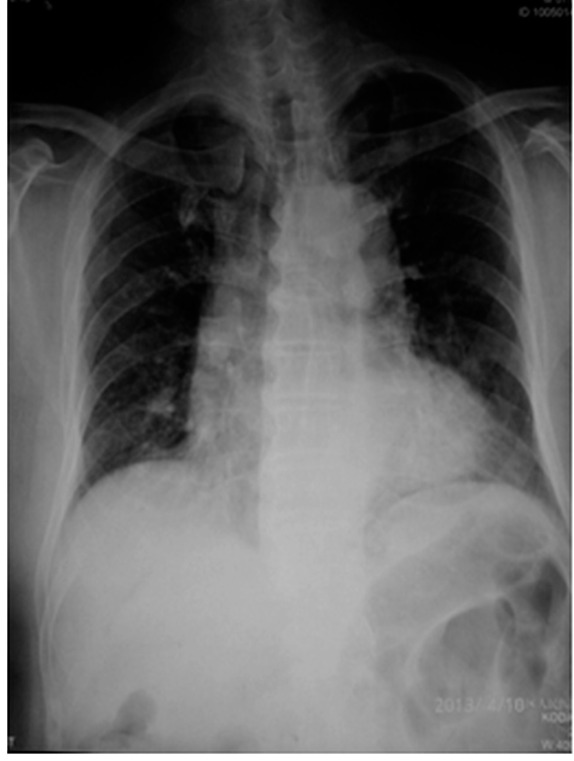
On April 3^rd^ 2013, the chest radiograph showed the left lower lung with a patchy, fuzzy shadow.

**Figure 2:**
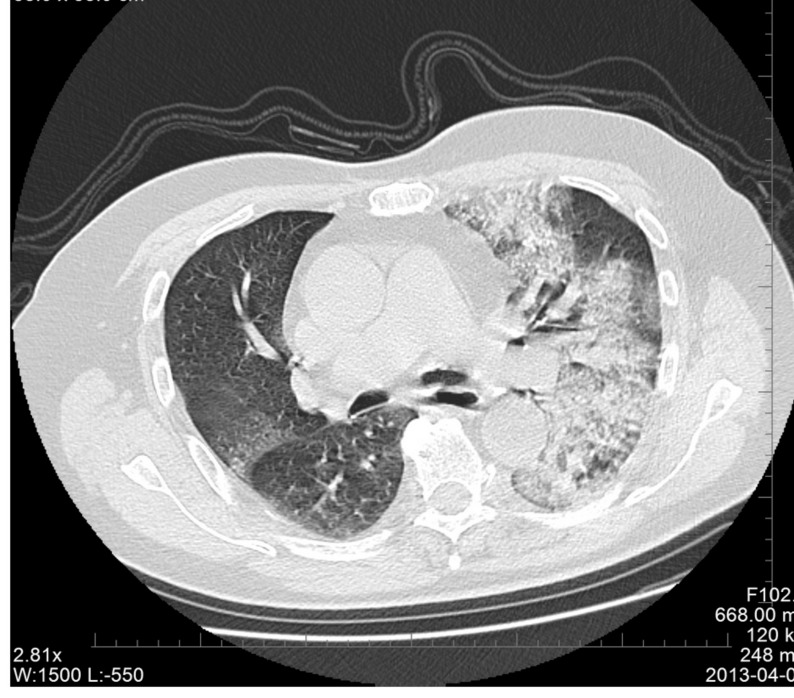
On April 7^th^ 2013, the chest computed tomography scan revealed the left lower lung with patchy inflammation.

A male patient, aged 77 years, was admitted to the Putuo District People’s Hospital of Shanghai City (Shanghai, China) after presenting with a fever for four days. On April 3^rd^ 2013, the patient experienced chills and fever, without evident cause or regularity, with a maximum temperature of 39.4°C. The patient did not suffer from a cough, expectoration, sore throat, runny nose, chest tightness, chest pain, pant or whole muscle and joint ache. A chest radiograph, obtained in the initial hospital, revealed a fuzzy shadow in the left lower lung (Fig. 1). After intravenous (i.v) treatment with ceftriaxone (2 g/day) and levofloxacin (0.5 g/day) for three days, the fever improved. The patient was admitted to the Jingnan District Centre Hospital of Shanghai (Shanghai, China) with a body temperature of 37.8°C. Emergency blood tests revealed a leukocyte count of 5×10^9^/l (neutrophils, 83.6%) and a C-reactive protein level of 192 mg/l. A chest computed tomography scan (Somatom Definition AS+ 128 Multi-Slice CT Scanner; Siemens, Munich, Germany) revealed a ‘frosted glass’ appearance in both lungs, and a high density shadow was observed in the left lower lobe (Fig. 2). In addition, a bronchiologram revealed inflammation and left lung consolidation. Since the patient was suspected of having pneumonia, the patient was admitted to the Central Hospital of Jingan District for further diagnosis and treatment. This study was conducted in accordance with the Declaration of Helsinki, and with approval from the Ethics Committee of the Central Hospital of Shanghai Jingan District. Written informed consent was obtained from the relatives of the patient.

### Past medical history

The patient had >30 years history of paroxysmal atrial fibrillation, and had been treated with amiodarone (0.2 g/day, orally). In addition, the patient had >20 years history of hypertension, up to a maximum of 150/90 mmHg; thus, amlodipine (5 mg every day) treatment had been used to control the blood pressure, which was determined to be of proper control. The patient denied history of diabetes, coronary heart disease, chronic bronchitis, asthma and chronic kidney disease, viral hepatitis, tuberculosis, typhoid fever and infectious disease.

### Physical examination

Physical examination revealed a body temperature of 39.1°C, a pulse of 98 bpm, a respiratory rate (RR) of 22 and a blood pressure of 130/70 mmHg. The patient was clear in mentality, had regular respiration, was cooperative on examination and their walking was not affected. The skin and mucosa exhibited no rash or hemorrhagic spots, and the lip mucosa had no cyanosis. Pharyngeal congestion was mild and the double tonsils were not enlarged. Breath sounds of the bilateral lung were coarse, and scattered rales were heard in the left lung, but without wheezing rale and pleural friction sounds. The heart rate of the patient was 98 bpm. Cardiac rhythm was regular, and there were no marked pathological murmurs heard in each valve area. The abdomen was soft, with no muscle tension, tenderness or rebound tenderness. No organomegaly or masses were observed, and the liver and spleen were not palpated under the ribs. There was no edema observed in the lower extremities, and the four limbs exhibited normal muscle force and muscle tension.

### Auxiliary examination

Hospital emergency blood gas analysis revealed a pH of 7.49, a PaCO_2_ of 38.6 mmHg, a PaO_2_ of 52.6 mmHg and a SaO_2_ of 87% (without oxygen). An electrocardiogram (ECG) demonstrated sinus rhythm, atrial premature beats, poor R wave progression in the anterior wall and T wave changes.

### Initial treatment and diagnosis

**Figure 3:**
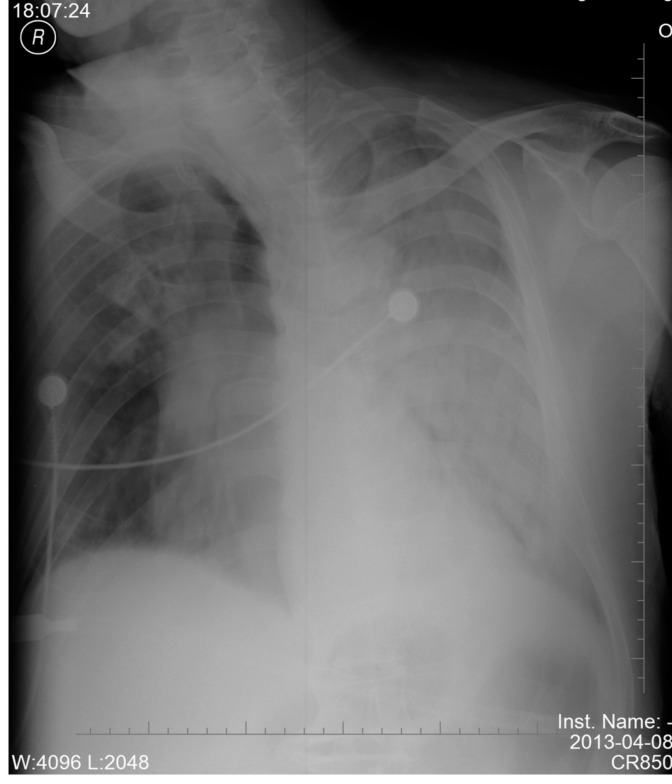
On April 8^th^ 2013, the chest radiograph showed the rapid progression of the lung lesions, with two pulmonary diffuse ground glass opacities.

Considering the initial diagnosis of severe pneumonia complicated with type I respiratory failure, the patient was administered oxygen therapy and methylprednisolone to reduce the systemic inflammatory response, and biapenem (0.6 g twice daily, i.v) and azithromycin (0.5 g/day, i.v) were applied as anti-infective agents. However, the patient continued to suffer from a fever with a body temperature of up to 39.1°C, experiencing distress on day 2 following admission. With repeated inquiries into the medical history of the patient, it was found that the patient had come into contact with chickens two weeks previously. Combining the epidemiological history and the rapid progression in the pulmonary lesions of the patient (Fig. 3), a diagnosis of human infection with H7N9 avian influenza was considered. Subsequently, the patient was isolated, administered active anti-infective agents (biapenem and azithromycin), an antiviral (oseltamivir; 75 mg/day, orally) and anti-inflammatory drugs (methylprednisolone at 200 mg/day). In addition, a biphasic positive airway pressure (BiPAP) ventilator was used for ventilatory support, and the patient received nasogastric enteral nutrition liquid. The case was reported to the Jingnan District Center for Disease Control (Shanghai, China) for nasopharyngeal swabs and blood sampling. In the early morning of April 9^th^, a diagnosis of severe human infection with H7N9 avian influenza was confirmed (1).

### Diagnosis confirmation

RNA was extracted from the throat-swab samples using the QIAamp Viral RNA Mini kit (Qiagen, Hilden, Germany), according to the manufacturer’s instructions. Specific RT-qPCR assays were performed to assess the presence of seasonal influenza viruses (H1, H3, or B), H5N1, severe acute respiratory syndrome coronavirus and novel coronavirus. RT-qPCR assays with self-designed specific primer and probe sets were subsequently performed for the detection of H1 to H16 and N1 to N9 subtypes, in order to verify the viral subtypes.

### Disease progression

**Figure 4:**
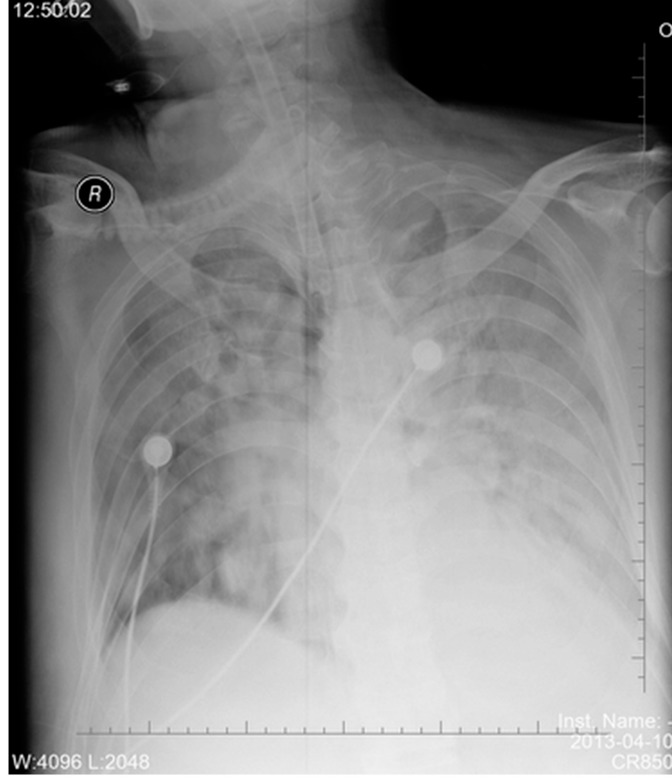
On April 10^th^ 2013, the chest radiograph showed the two pulmonary diffuse ground glass opacities, with a degree of fusion into nodules.

Following hospital admission, the body temperature of the patient fluctuated between 39 and 40°C, with shortness of breath further aggravating. At 18:00 on April 9^th^, the patient presented with cyanosis of the lips and limbs. In addition, the fingertip pulse oximeter monitor displayed a SPO_2_ of 45%, and the patient underwent an emergency tracheal intubation for assisted ventilation (BIPAP mode; set inspiratory pressure, 20 cm H_2_O; positive end expiratory pressure, 10 cm H_2_O; assisted spontaneous breathing, 10 cm H_2_O; inspiratory time, 1.3 sec; respiratory rate, 16 times/min; concentration of oxygen inhalation, 100%), while improving microcirculation and anti-leakage. On April 10^th^, the patient exhibited a damaged liver performance, and was administered nutritional support treatment (human albumin), intravenous immunoglobulin to strengthen the immunity, drugs for liver protection [Coenzyme Q10 (20 mg orally, three times daily); and Compound Glycyrrhihizin (0.2 g i.v. once daily)], daily fluid therapy [5% glucose solution (500 ml), 5% glucose and sodium chloride solution (500 ml) and 0.9% sodium chloride solution (500 ml)], and psychological treatment. The chest radiograph showed progression of the lesions in the right lung (Fig. 4). In addition, blood biochemical analysis revealed glucose levels of 16.8 mmol/l, myoglobin levels of 1,483 ng/ml, creatine kinase levels of 1,561 U/l, lactate dehydrogenase levels of 1,007 U/l, albumin levels of 20 g/l, alanine aminotransferase levels of 82 IU/l, aspartate aminotransferase levels of 185 IU/l, urea nitrogen levels of 15.85 mmol/l, creatinine levels of 152.50 μmol/l, potassium levels of 4.47 mmol/l, sodium levels of 155 mmol/l and chloride levels of 115.10 mmol/l. Plasma osmotic pressure was 335.74 mmol/l, which was calculated from the following formula: 2 × (serum sodium concentration + serum potassium concentration) + blood glucose concentration; the normal reference value is between 280 and 310 mmol/l. Considering the hypertonicity of the plasma, the patient was administered cold boiled water, an aggressive diuretic and a small dose of dopamine treatment (5 μg/kg/min, i.v). If the serum creatinine level had continued to increase and the urine volume decrease, continual renal replacement therapy at the bedside was considered. On April 10^th^ at 15:00, a small amount of coffee colored material emerged from the corner of the lips, and a 50-ml volume of the coffee colored substance was drained. Following application of gastrointestinal decompression, an occult blood test of the vomit showed occult blood++, indicating a moderate degree of microscopic hematuria. The patient was fasted, gastrointestinal decompression was continued and proton pump inhibitor treatment was applied. By 23:00, the patient had a rapid heartbeat and shortness of breath. The monitor displayed the heart rate at 150 bpm, the RR at 35 and an SPO_2_ of 87%. In addition, the ECG revealed atrial fibrillation at a rapid ventricular rate. The patient was immediately administered 200 mg Cordarone, 20 mg furosemide, 40 mg Xinkang, 50 mg morphine and 0.4 mg cedilanid intravenously. After 1 h, the heart rate and breathing of the patient improved. On April 13^th^, the patient exhibited a persistent fever, circulation failure with renal failure. Furthermore, the blood pressure was unable to be maintained and the levels of urine were very low. On April 14^th^ at 00:55, the patient succumbed to multiple organ failure, which caused cardiac arrest, and subsequently the loss of ventilator breathing, an arterial pulse and a pupillary light reflex. The ECG was shown to be asystole, and clinical death was confirmed.

## Discussion

Severe human infection with H7N9 avian influenza progresses very rapidly. Since no specific and effective therapy has been developed, treatment is difficult. Early detection and diagnosis, which may slow the disease progression to prevent severe pneumonia, are essential for improving patient outcome. However, it also should be considered that the most effective approach to managing the problem of H7N9 viral infection is to educate the general public on aspects of healthcare, such as self-prevention and the promotion of basic sanitation, which are associated with the transmission of respiratory infections (4).

Epidemiological history is one of the main clues to the diagnosis of infectious disease; however, a latency that is longer than the conventional report or no clear epidemiological contact history should not be considered as exclusion criteria for diagnosis. Detection of the H7N9 virus in samples collected from a pigeon and chickens at a market in Shanghai was confirmed by the China Animal Disease Control Center (Beijing, China) (5). Severe infection with H7N9 avian influenza usually has the initial symptoms of fever and respiratory system infection, and monitoring of the sustained clinical symptoms and dynamic radio-imaging should be performed. In addition, samples should be collected immediately following admission for virus nucleic acid detection to confirm the diagnosis, which may promptly improve the medication time (6). The influenza virus not only invades the respiratory system, but also affects multiple organs. With the added complications of the different baseline statuses of patients and the atypical clinical manifestations, dynamic monitoring of pulmonary imaging is particularly important for the early identification of lung infections and diseases, since viral pneumonia characteristic changes can be detected. In addition, close monitoring of blood gas analysis, liver and kidney function, myocardial enzymes and immune indexes should be performed, as this may lead to early detection of the disease and subsequent prevention of complications. For cases that cannot be clinically confirmed, if there are underlying diseases and risk factors, timely administration of oseltamivir should be provided to inhibit viral replication, as well as treatments aimed at the causes. The emergence of the novel H7N9 influenza has caused global concern with regard to the ability of this virus to spread between humans (7); however, until now, there is no sufficient evidence of that which requires further study.

In addition, attention should be paid to the control of complications, nutritional support, reconstruction and stabilization of the internal environment and immune homeostasis. Early administration of glucocorticoids can inhibit the inflammatory reaction, reduce the release of cytokines and inflammatory mediators, and reduce alveolar exudation. Furthermore, timely trachea intubation and application of respiratory support technology can correct hypoxia, protect major organ functions and prevent multiple organ dysfunction (8). For patients with renal insufficiency, continuous hemofiltration applied at the bedside may be the primary method for improving the prognosis.

In conclusion, severe human infection of H7N9 avian influenza often involves multiple systems, with rapid progression and poor prognosis, emphasizing the requirement for multidisciplinary, comprehensive management. Airway management, prevention of infection, mechanical ventilation, water balance support, electrolyte and acid-base balance, nutritional support and the maintenance of important organ function are key to a successful outcome for critically ill patients (9). In addition, in elderly patients, the pre-existence of a disease may be a risk factor, and may directly affect the prognosis of the patients (10).
